# Simulation-Based Analysis of Trial Design in Regional Anesthesia

**DOI:** 10.1155/2024/6651894

**Published:** 2024-03-15

**Authors:** Melisa Pasli, Dmitry Tumin, Ryan Guffey

**Affiliations:** ^1^Brody School of Medicine, East Carolina University, Greenville, NC, USA; ^2^Department of Pediatrics, Brody School of Medicine, East Carolina University, Greenville, NC, USA; ^3^Department of Academic Affairs, Brody School of Medicine, East Carolina University, Greenville, NC, USA; ^4^Department of Anesthesiology, Washington University School of Medicine, Saint Louis, MO, USA

## Abstract

**Background:**

In regional anesthesia, the efficacy of novel blocks is typically evaluated using randomized controlled trials (RCTs), the findings of which are aggregated in systematic reviews and meta-analyses. Systematic review authors frequently point out the small sample size of RCTs as limiting conclusions from this literature. We sought to determine via statistical simulation if small sample size could be an expected property of RCTs focusing on novel blocks with typical effect sizes.

**Methods:**

We simulated the conduct of a series of RCTs comparing a novel block versus placebo on a single continuous outcome measure. Simulation analysis inputs were obtained from a systematic bibliographic search of meta-analyses. Primary outcomes were the predicted number of large trials (empirically defined as *N* ≥ 256) and total patient enrollment.

**Results:**

Simulation analysis predicted that a novel block would be tested in 16 RCTs enrolling a median of 970 patients (interquartile range (IQR) across 1000 simulations: 806, 1269), with no large trials. Among possible modifications to trial design, decreasing the statistical significance threshold from *p* < 0.05 to *p* < 0.005 was most effective at increasing the total number of patients represented in the final meta-analysis, but was associated with early termination of the trial sequence due to futility in block vs. block comparisons.

**Conclusion:**

Small sample size of regional anesthesia RCTs comparing novel block to placebo is a rational outcome of trial design. Feasibly large trials are unlikely to change conclusions regarding block vs. placebo comparisons.

## 1. Introduction

Systematic reviews and meta-analyses of randomized controlled trials (RCTs) are considered the pinnacle of evidence in clinical medicine [1–3]. When properly executed, meta-analyses can yield robust findings that otherwise could only be obtained through large RCTs. Such large RCTs may be infeasible due to regulatory, funding, or logistical considerations [4–8]. In the absence of definitive large trials, meta-analyses of smaller trials are likely to continue shaping clinical practice. However, meta-analyses can be limited by the sample size of constituent studies, and underpowered meta-analyses may fail to yield conclusive results [9, 10]. Conversely, meta-analyses of small studies could also arrive at excessively optimistic positive results, which might be refuted if a large trial were conducted [6]. Therefore, understanding the reasons for the proliferation of small RCTs can inform trial design, peer review, and research funding priorities.

In regional anesthesia, evaluation of novel block techniques has generated a large number of small RCTs comparing new blocks to placebo, sham block, or no block. Authors of systematic reviews in this area have frequently noted small sample sizes of included trials as a limiting factor to their conclusions [11–14]. Small trial sample sizes are variously attributed to publication pressure, limited funding, and logistical challenges of conducting multicenter trials [7, 8, 15, 16]. Specific to regional anesthesia, there may also be greater interest in studying new techniques presumed to be highly effective (implying low sample size on *a priori* power calculation) than studying smaller differences between clinically effective blocks, which would require larger sample sizes, but may be clinically irrelevant [11, 14, 17, 18].

The statistical relationship between a larger anticipated effect size and smaller planned sample size is well established [19]. However, the cumulative impact of this relationship on the conduct of large vs. small trials is not well defined. We propose that conducting numerous small trials may be a rational response of investigators primarily interested in identifying if novel nerve blocks are clinically effective. We hypothesize the overall evidence base in support of each block will be composed of primarily small RCTs, even if publication bias and limitations due to inadequate resources or incorrect sample size determination were eliminated entirely. Based on input values derived from recent regional anesthesia meta-analyses, we performed a simulation study to determine the number of large trials and patient enrollment which may be expected in the literature on a novel nerve block. Our secondary aim was to examine how these characteristics of a sequence of trials could be influenced by common statistical recommendations intended to improve trial design.

## 2. Methods

### 2.1. Rationale and Study Design

We simulated the conduct of a sequence of RCTs designed to test the performance of a novel block against a control group on a single continuous outcome measure (such as pain score or opioid consumption). This study did not involve human subject research and did not require institutional review board (IRB) approval. Inputs for our simulation analysis were obtained from a systematic bibliographic search of meta-analyses on established regional anesthesia techniques, which is detailed below. Briefly, we assumed that with the advent of each novel block, superiority trials would be conducted to compare its efficacy vs. a control group of placebo, sham block, or no block. Sham block refers to the needle being inserted and saline being injected while the patient is blinded. In the case of no block, the patient is not typically blinded.

After a certain number of RCTs have been conducted, we assumed that a meta-analysis would be published which would help inform effect size assumptions for future RCTs. We further assumed that additional meta-analyses would be published periodically, and that new RCTs would continue to be conducted until the literature was saturated, or until investigation of this novel block was curtailed due to lack of efficacy (quantification of these conditions is described below). We then calculated the expected proportion of RCTs conducted under these assumptions that would enroll a large sample (defined empirically based on our literature search), as well as the number of patients who would have been enrolled in the largest trial and the number of patients who would have been enrolled by the time of the final meta-analysis.

We used violin plots to represent simulated data as medians (white dots) with interquartile ranges (bars) across 1,000 simulated trial sequences. The width of the violin plot corresponds to the distribution of the values plotted vertically from the minimum to the maximum. Definitions and assumptions for our simulation model were based on a systematic bibliographic search of meta-analyses of major regional anesthesia techniques studied for at least 5 years. In June–September 2022, we used PubMed to search for each combination of block and indication listed in Supplemental Appendix 1. For each block and indication, we selected the most recent PubMed-indexed meta-analysis which compared one or more quantitative, continuous outcomes measuring acute pain (any pain-related outcome assessed up to 24 hours postoperatively) between any type of block and no block (including sham block or placebo). Meta-analyses including only categorical outcomes, only comparisons of different block types to one another, or study designs other than RCTs were excluded. Meta-analyses which primarily included pediatric patients were also excluded, due to greater challenges of enrolling large samples of children.

For each meta-analysis, we selected one focal outcome: the comparison of quantitative and continuous data between a block and no block (or placebo/sham block), for which the largest number of trials were reported in the results section. In the event of multiple eligible outcomes and comparisons being reported based on the same number of trials, we focused on the outcome that appeared first in the results section. In reference to this focal outcome, we extracted the effect size and trial sample size from each meta-analysis, as defined in Supplemental Appendix 2. From each publication, we also extracted the total number of trials comparing a given block to no block, placebo, or sham block.

### 2.2. Data Analysis

We used data from this bibliographic search to derive inputs (assumptions) for our simulation analysis (Table 1) and compared these assumptions to prior publications on trial design and systematic reviews in anesthesiology. [21–23] The standard deviation of the effect size was derived by calculating the half-width of the 95% confidence interval (CI) around the standardized mean difference, dividing this number by the critical *Z* statistic, which was approximated at 1.96, and taking the median of this output. The sample size of the first simulated RCT was set at 30 cases per group (a common trial sample size that is sufficient to apply standard statistical methods) [20]. Standardized effect sizes for each trial were assumed to be drawn from a normal distribution, and sample sizes for subsequent trials were assumed to be determined by power analysis. A minimum clinically relevant effect size (*d* = 0.11) and a threshold for what was considered a large trial (*N* = 256) were defined based on the bibliographic search. After the first RCT, we assumed subsequent trials would be powered to detect the smallest effect previously reported, or the minimal clinical effect, whichever was largest. Desired power and significance levels were set at common levels of 80% and *p* = 0.05, respectively. [21] The *p* values from the simulated meta-analyses were based on random-effects regression fitted using restricted maximum likelihood.

### 2.3. Assumptions

We assumed that after a certain number of trials, a meta-analysis would be conducted and that updated meta-analyses would be published as more trials were completed. Once a meta-analysis has been conducted, we assumed further trials would be powered based on the pooled effect size from the most recent meta-analysis. Finally, we assumed investigation of the novel block would be curtailed due to saturation of the literature after a certain number of trials were reached. Alternately, we assumed that investigation would be curtailed when a meta-analysis showed an effect size below the minimal clinically relevant effect threshold or when the sample size required for the next planned RCT became prohibitively large (*N* = 1,000, reflecting an exceptionally large sample size for RCTs in regional anesthesia).

### 2.4. Statistical Parameters

**Figure 1:**
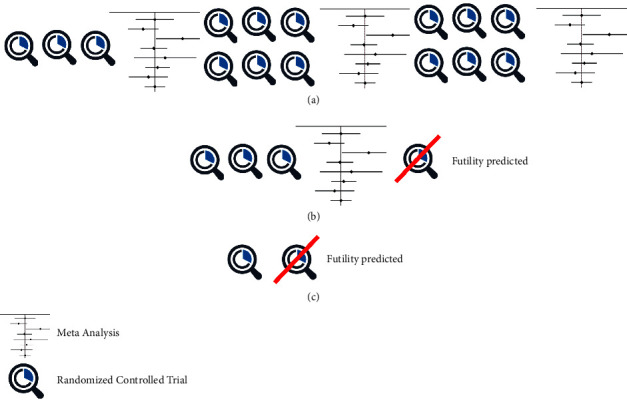
Subset of 1000 simulated randomized sequences. (a) Complete sequence (b) incomplete sequence terminated after first meta-analysis due to futility (c) incomplete sequence terminated after first trial due to futility. The size of each subsequent trial is based on a power analysis from the previous trial or meta-analysis. Futility was inferred when the size of the next trial in sequence would have been over the predetermined limit of 1000 cases. The results of each trial are simulated based on a random draw from a normal distribution around the prespecified effect size.

Possible sequences of simulated trials and meta-analyses are illustrated in Figure 1. The size of each subsequent trial is based on a power analysis from the previous trial or meta-analysis. Futility was inferred when the size of the next trial in the sequence was over the predetermined limit of 1000 cases. In addition to the baseline scenario (defined in Table 1), we separately examined the impact of several statistical recommendations intended to improve trial design. First, we considered the proposal from the American Statistical Association to decrease the statistical significance threshold from *p* < 0.05 to *p* < 0.005 (lower alpha) [24]. Second, we considered common guidance to select a higher power threshold, specifically 90% as compared to 80% (higher beta) [19]. Third, we considered the recommendation to increase trial sample size by 15% to account for common outcomes (including pain scores and opioid use) being non-normally distributed [25]. Under each condition, we simulated 1,000 trial sequences and described our study outcomes (number of large trials; number of patients in the largest trial; number of total patients enrolled) using medians and interquartile ranges (IQR) across the 1,000 simulations.

While our primary interest was in studying the conduct of trials intended to demonstrate the large effect of a new type of block, we also repeated our analyses for the case of comparing block vs. block, where we assumed the average difference in outcomes would be of marginal clinical significance (defined as the upper threshold of a “small” effect size, *d* = 0.34) [22]. For context, an effect size of *d* = 0.34 would rate as the third-smallest of the effect sizes extracted from our literature search (Supplemental Appendix 1). Data analysis was conducted using Stata/16.1 SE (College Station, TX: StataCorp., LP), with the simulation program code included in Supplemental Appendix 3.

### 2.5. Sequence of RCTs until Saturation

To illustrate the progression of a simulated sequence of RCTs (Figure 1(a)), suppose the initial pilot trial (*N* = 60) shows a standardized effect size of *d* = 0.52 (moderately clinically significant effect). The second trial, powered for this effect, would require a sample size of *N* = 120 and shows a stronger difference of *d* = 1.31. This stronger difference was simulated based on a random draw from a normal distribution of effect sizes and was not assumed to have any specific underlying cause. The third trial, still powered for the smallest effect size seen to date (*d* = 0.52, implying *N* = 120), shows a very weak effect, *d* = 0.29. Therefore, the fourth trial would be conducted with *N* = 388 patients (exceeding our definition of a “large” trial) and would find *d* = 0.76. At this point, a meta-analysis of the first 4 trials would reveal a pooled effect size of *d* = 0.72, meaning the next six trials would be conducted with *N* = 62 patients each. The second interim meta-analysis would still demonstrate a clinically significant pooled effect (*d* = 0.70), meaning another six trials would be conducted with *N* = 68 each. At this point, the literature would be considered to have been saturated, with a final pooled effect size of *d* = 0.78.

## 3. Results

**Figure 2:**
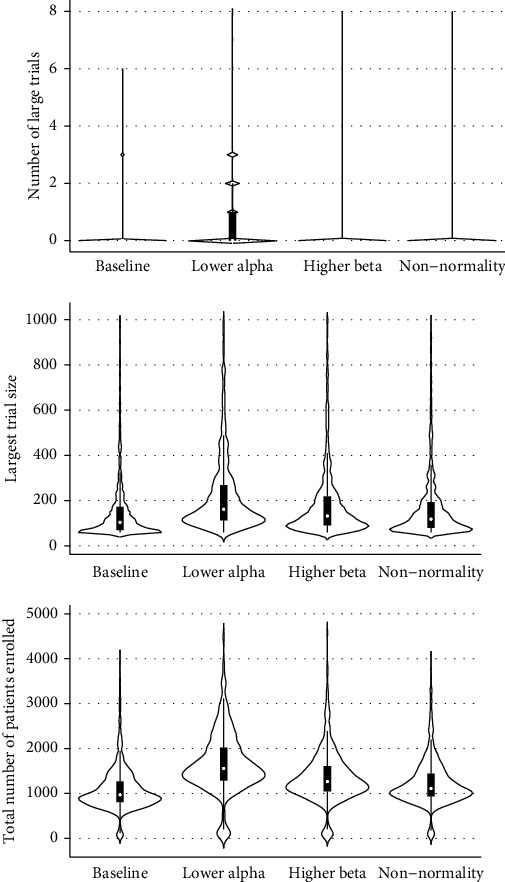
Sample size of largest trial and total sample size of all trials for simulated randomized controlled trials of a novel block vs. no block. Data are shown as medians (bars) with interquartile ranges (lines) across 1,000 simulated trial sequences.

The median trial sequence across 1,000 simulations was predicted to contain no large trials (IQR: 0, 0) and was predicted to have a maximum trial size of just 104 patients (IQR: 70, 172), with a total of 970 patients recruited across all trials in the sequence (IQR: 806, 1269).  Figure 2 illustrates how these expected outcomes could change with implementation of each of the statistical recommendations summarized above. Decreasing the statistical significance threshold to *p* < 0.005 would have the most profound impact on increasing the size of the largest trial from 104 to 162 and the total number of patients enrolled from 970 to 1556. Increasing statistical power from 80% to 90% and inflating the sample size by 15% to account for non-normally distributed data had similar but weaker effects on total patient enrollment. Under all conditions, the median RCT sequence contained no large trials and was terminated after 16 trials due to saturation of the literature. Final effect sizes and their *p* values under each condition are shown in Table 2, with the median effect size in each case approaching the preset value of *d* = 0.77.

**Figure 3:**
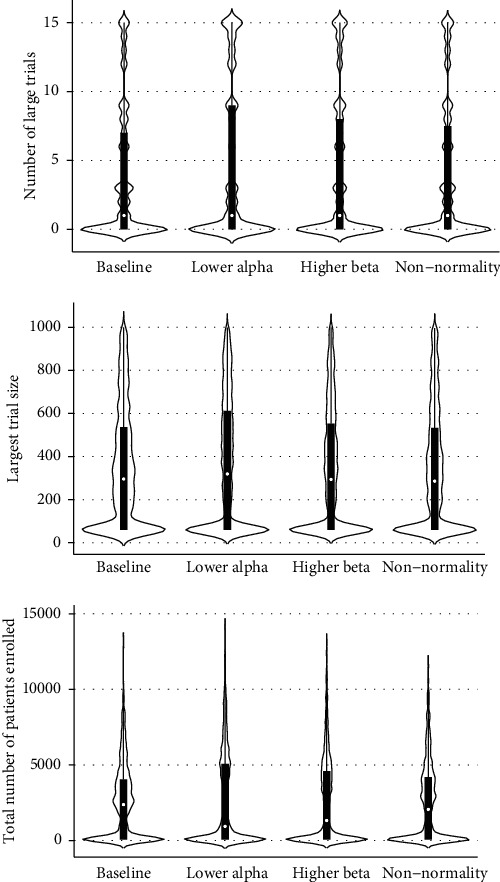
Number of large trials (*N* ≥ 256), sample size of largest trial, and total sample size of all trials for simulated randomized controlled trials comparing two hypothetical blocks to one another. Data are shown as medians (bars) with interquartile ranges (lines) across 1,000 simulated trial sequences.

In Figure 3, we summarize results from our secondary analysis, where we simulated comparison of two blocks to one another. With our initial simulation inputs, we found that in this case, the median sequence of trials would contain 1 large trial (IQR: 0, 7). Decreasing the alpha level or increasing power had no effect on the predicted number of large trials but tended to reduce the median number of all trials per sequence from 16 to 3 and 4, respectively, reflecting curtailment of block-*versus*-block comparisons due to statistical futility. This paradoxical result may be explained by our assumption that very large (*N* > 1000) trials would be infeasible to conduct. When statistical assumptions for sample size determination are made more stringent, this threshold tends to be reached earlier in the sequence of trials comparing two blocks of similar efficacy. Notably, early curtailment of trial sequences in the block-*versus*-block scenario frequently led to an inability to reject the null hypothesis at the final meta-analysis (e.g., upper quartile of the final effect size *p* value was >0.500 in each scenario, Table 2). In our simulation, this represents a Type II error, since a nonzero population effect size was predetermined by our approach.

## 4. Discussion

A lack of large RCTs has been identified as a specific source of concern in regional anesthesia meta-analyses [11–14]. Our simulation analysis sought to quantify expected patient enrollment and the number of large trials in the literature on a novel block, demonstrating that if RCTs of a new block are conducted based on conventional statistical guidelines until the literature reaches saturation, the largest trial would likely enroll just over 100 patients, far below the “large” trial threshold (*N* ≥ 256). Therefore, the absence of large trials in regional anesthesia meta-analyses may be primarily related to statistical properties of the conventional approach to trial design rather than author- or institution-level biases. This statistical tendency is compounded by limited resources to conduct RCTs and a potential lack of interest in pursuing larger trials of blocks already known to be effective in clinical practice.

Putting this analysis into context, our bibliographic search demonstrated that meta-analyses comparing block versus no block or placebo tended to include few trials (highest number of trials = 25), and these trials tended to have limited sample sizes (range: 18, 378 patients). Furthermore, the evidence base for many of the blocks listed in Supplemental Appendix 1 often consisted of just a few studies comparing them to no block or placebo; we could not identify any eligible meta-analyses of RCTs for 5 of the 29 nerve blocks we had considered. This sparsity of literature may reflect the common use of certain blocks in clinical practice without requiring high-level evidence to verify their efficacy. For example, practice has evolved to favor serratus anterior blocks over paravertebral blocks in breast surgery despite lacking large trials demonstrating superiority due to easier performance and equivocal analgesic efficacy [12]. Paravertebral blocks are more challenging to perform and invasive, and have potential for severe risks including epidural spread, pneumothorax, and epidural hematoma [12, 26]. As such, lower levels of evidence are required to convince practitioners to switch from higher risk, older, more challenging procedures to newer, more superficial, ultrasound-guided procedures.

Conclusions from our simulation analysis go beyond the textbook definition of statistical power by considering how meta-analyses, perceived futility, and saturation of the literature contribute to the cumulative evidence base forming around a novel block. Our bibliographic search, used to establish input values for the simulations, returned very similar inputs to previous reviews of meta-analyses in anesthesiology. For example, our assumed mean population effect size of 0.77 was very close to the median effect size of 0.80 demonstrated in a prior systematic review [21], and our assumed number of trials required to reach literature saturation was consistent with a prior systematic review, in which the median meta-analysis contained 16 trials [23]. Following the suggestion by the American Statistical Association [24], our results demonstrated that reducing the statistical significance threshold (alpha) had the biggest impact at increasing the sample size of the largest trial and the total sample size of all trials in a sequence. However, larger trials are unlikely to change conclusions if a comparison has a moderate to large effect size and are not required to demonstrate the efficacy of most regional anesthetics relative to placebo. Factors other than efficacy, such as safety and ease of performance, may be more important when choosing between two effective regional anesthetics, but these are rarely selected as primary endpoints for RCTs, and would not typically inform sample size determination.

Our simulation analysis was subject to limitations based on how it accounted for certain features of the clinical research process. First, we deliberately chose not to simulate the impact of publication bias, missing or inaccurate power analysis [21], or questionable research practices in the clinical trials literature. We set the upper limit of the trial sample size at a generous *N* = 1,000 to capture the low chance of large RCTs being conducted even when resources were relatively unlimited. However, we assumed that investigators would easily be able to access data from past trials and that they would defer to the effect size from the most recent meta-analysis when powering a new trial (vs. using pilot trial data from their own institution or using observational data). We also did not consider categorical outcomes, which tend to require larger sample sizes, and, perhaps for this reason, are rarely selected as primary trial endpoints in regional anesthesia. Lastly, we did not account for the multiplicity of outcomes that could be evaluated (e.g., 2-hour vs. 4-hour pain scores), which could contribute to continued growth in the number of trials as different trials seek to evaluate different aspects of the efficacy or safety of a novel block.

In sum, our simulation analysis addresses a recurrent critique of small RCT size in regional anesthesia systematic reviews. We demonstrate that under conditions of perfect information, rigorous trial design, and near-unlimited resources, small RCTs would remain the standard in research comparing novel nerve blocks to placebo, sham, or no block. Furthermore, we demonstrate that adoption of recommended statistical practices, such as reducing the alpha level or increasing trial power, would have limited impact on increasing the number of large trials when comparing a novel block vs. no block, while decreasing the total number of trials that compare different blocks to one another. The latter, paradoxical result would be due to the likely statistical futility of comparisons among multiple effective blocks. Registry-based analysis could provide valuable pragmatic clinical and safety data that may be lacking even in large RCTs. It would be worthwhile to ensure large surgical registries collect sufficient data to meaningfully compare the immediate and longer-term risks and benefits of specific regional anesthesia techniques. Meanwhile, peer review and meta-analysis of RCTs in regional anesthesia should consider both logistical and statistical reasons for limited sample sizes and avoid setting unrealistic expectations for the conduct of large trials that are unlikely to change practice.

## Figures and Tables

**Table 1:** Input data for simulation analyses, based on published meta-analyses of nerve blocks.

Input parameter	Definition	Value	Comments
Starting trial sample size	Sample size sufficient for standard statistical techniques	30	As recommended in a prior publication [20]
Large trial sample size	90^th^ percentile of largest trial size included in published meta-analyses	256	Rounded up to nearest multiple of 2
Population effect size	Median absolute effect size among published meta-analyses	0.77	On a previous systematic review, median effect size for quantitative outcomes was 0.8 [21]
SD of effect size	Median of the 95% CI half-width divided by 1.96	0.27	Similar to previously published IQR of effect sizes for quantitative outcomes (0.6–1.0) [21]
Minimum effect size deemed clinically relevant	10^th^ percentile of the absolute effect sizes among published meta-analyses	0.11	0.10–0.34 is considered a small effect size; 0.35–0.64 is considered a medium effect size; 0.65–1.19 is considered a large effect size [22]
Number of trials needed for earliest meta-analysis	10^th^ percentile of the number of trials among published meta-analyses	4	
Number of trials needed for next meta-analysis	SD of number of trials, among published meta-analyses	6	
Number of trials needed to reach saturation of the literature	90^th^ percentile of the number of trials among published meta-analyses	16	On a previous systematic review, the median meta-analysis included 16 trials (IQR: 10, 28) [23]

CI, confidence interval; IQR, interquartile range; SD, standard deviation.

**Table 2:** Median and interquartile range (IQR) of final effect size and its *p* value across 1,000 simulated sequences of randomized controlled trials.

Scenario	Median effect size	IQR of median effect size	Median *p* value	IQR of median *p* value^*∗*^
Block vs. no block, baseline	0.78	0.73, 0.83	<0.001	<0.001, <0.001
Block vs. no block, lower alpha	0.78	0.73, 0.83	<0.001	<0.001, <0.001
Block vs. no block, higher power	0.78	0.73, 0.82	<0.001	<0.001, <0.001
Block vs. no block, assumed non-normality	0.78	0.73, 0.83	<0.001	<0.001, <0.001
Block vs. block, baseline	0.30	0.12, 0.37	<0.001	<0.001, 0.509
Block vs. block, lower alpha	0.24	0.13, 0.36	0.082	<0.001, 0.505
Block vs. block, higher power	0.27	0.11, 0.37	0.036	<0.001, 0.558
Block vs. block, assumed non-normality	0.28	0.12, 0.37	0.003	<0.001, 0.533

^*∗*^ *P* values of ≤0.05 were deemed statistically significant. IQR, interquartile range.

## Data Availability

The data used to support the findings of this study are included in the manuscript.

## Supplementary Materials

Supplementary Materials Appendix Table 1: combinations of block type and indication included in search of regional anesthesia meta-analyses, and characteristics coded from each article. Appendix 2: data elements coded from each meta-analysis article. Appendix 3: Stata program to simulate sequence of randomized controlled trials.
