# Supplementary material for: Simulation-Based Analysis of Trial Design in Regional Anesthesia
Source: Anesthesiol Res Pract. 2024 Mar 15;2024:6651894. doi: 10.1155/2024/6651894 (PMC10959581; doi:10.1155/2024/6651894)
Supplement: Supplementary Materials — Appendix Table 1: combinations of block type and indication included in search of regional anesthesia meta-analyses, and characteristics coded from each article. Appendix 2: data elements coded from each meta-analysis article. Appendix 3: Stata program to simulate sequence of randomized controlled trials. [file 6651894.f1.zip › Supplemental Appendix 3.docx]

**Appendix 3.** Stata program to simulate sequence of randomized controlled trials.

capture program drop simseq // overwrite existing program name, if applicable

program define simseq, rclass // define program syntax

version 16.0

syntax , [seed(int 1)] /// starting seed to replicate results

[ITERate(int 10)] /// number of iterations (default to 10)

delta(real) /// hypothesized effect size

sd_delta(real) /// SD of effect size (across trials)

large(integer) /// threshold for large trial

meta_start(int) /// starting # of trials for meta-analysis

meta_iter(int) /// iterating # of trials for meta-analysis

meta_sat(int) /// number of trials needed to reach saturation

mineffect(real) /// minimum clinically significant effect size

[n_start(int 60)] /// starting sample size (default=30/group)

[alpha(real .05)] /// alpha level

[beta(real .8)] /// beta-level

[maxsize(int 1000)] /// maximum feasible N (assumed 1000)

[nnormal] // add 15% to sample size if assuming non-normality

/* load input parameters */

global delta = `delta'

global sd = `sd_delta'

global X = `meta_start' + 1

global Y = `meta_iter'

global Z = `meta_sat'

global N = `n_start'

global M = `mineffect'

global I = `iterate'

global large = `large'

global A = `alpha'

global B = `beta'

global NN = ("`nnormal'"=="nnormal")

global MAX = `maxsize' // max trial size

set seed `seed'

preserve

forvalues iter = 1/$I {

qui clear

qui set obs 1

local exit = 0

qui gen n = $N // total N

qui gen effect = rnormal($delta,$sd)

qui gen n1 = n/2

qui gen n2 = n/2

qui gen sd1 = 1

qui gen sd2 = 1

qui gen m1 = 1

qui gen m2 = 1+effect

local rct = 1

while `rct' < $Z & "`exit'" ~= "1" {

local ++rct

local exit = 0

if `rct'<$X {

qui sum effect if effect>=$M

capture local mineffect = r(min)

if `mineffect'==. {

local mineffect = $M // assume MCD

}

local mineffect = `mineffect' + 1

}

if `rct' >= ($X) ///

& floor((`rct'-$Y-$X) / $Y )==((`rct'-$Y-$X) / $Y ) {

qui meta esize n1 m1 sd1 n2 m2 sd2, esize(mdiff)

qui meta sum, iter(1) // do not estimate t2

local mineffect = -1 * r(theta) + 1

// supersedes above calculation

if `mineffect' < $M local exit = 0.5

// get ready to exit

}

if `exit' != 1 {

qui power twomeans 1 `mineffect', power($B) alpha($A)

local n = r(N)

if $NN==1 local n = ceil((1.15 * `n'))

if (`n'/2) != ceil(`n'/2) local n = `n' + 1

if `n' <= $MAX {

// must be below max trial size

qui set obs `rct'

qui replace n = `n' in `rct'

qui replace effect = ///

rnormal($delta,$sd) in `rct'

qui replace n1 = (n/2) in `rct'

qui replace n2 = (n/2) in `rct'

qui replace sd1 = 1 in `rct'

qui replace sd2 = 1 in `rct'

qui replace m1 = 1 in `rct'

qui replace m2 = 1+effect in `rct'

}

}

if `exit' == 0.5 local exit = 1 // exit now

if `n' > $MAX local exit = 1 // exit now

}

/* final meta-analysis */

qui meta esize n1 m1 sd1 n2 m2 sd2, esize(mdiff)

qui meta sum, iter(1) // do not estimate t2

local finaleffect = r(theta)

local finaleffect = -1*`finaleffect'

qui count if n>=$large & n<.

local numlarge = r(N)

qui count

local numtotal = r(N)

qui egen pttotal = total(n)

qui sum pttotal

local pttotal = r(max)

qui sum n

local maxsize = r(max)

/* save results to matrix */

matrix output = ///

[`numtotal',`numlarge',`maxsize',`pttotal',`finaleffect']

if `iter'==1 matrix O = output

if `iter'>1 matrix O = O\output

}

qui clear

local maxcol = colsof(O)

qui svmat O

forvalues col = 1/`maxcol' {

qui _pctile O`col', p(25 50 75)

local O`col'_lcl = r(r1)

local O`col'_median = r(r2)

local O`col'_ucl = r(r3)

matrix O`col' = [`O`col'_median' , `O`col'_lcl', `O`col'_ucl' ]

if `col' == 1 matrix R = O`col'

if `col' > 1 matrix R = R \ O`col'

}

matrix rownames R = Ntrials Nlarge maxsize totalpt finalD

matrix colnames R = median p25 p75

matrix list R

return matrix SEQ = R

restore

end // end program syntax

/* example of program run with inputs for baseline scenario,

comparing block vs. no block */

simseq, delta(0.77) sd_delta(.27) large(256) ///

meta_start(4) meta_iter(6) meta_sat(16) ///

mineffect(.11) iter(1000) seed(8)
