# Supplementary material for: Simulation-Based Analysis of Trial Design in Regional Anesthesia
Source: Anesthesiol Res Pract. 2024 Mar 15;2024:6651894. doi: 10.1155/2024/6651894 (PMC10959581; doi:10.1155/2024/6651894)
Supplement: Supplementary Materials — Appendix Table 1: combinations of block type and indication included in search of regional anesthesia meta-analyses, and characteristics coded from each article. Appendix 2: data elements coded from each meta-analysis article. Appendix 3: Stata program to simulate sequence of randomized controlled trials. [file 6651894.f1.zip › Supplemental Appendix Table 1.docx]

**Appendix Table 1.** Combinations of block type and indication included in search of regional anesthesia meta-analyses, and characteristics coded from each article.

-

| **Block and indication** | **Citation** | **Number of trials comparing block to control** | **Focal comparison** | **Focal outcome** | **Trial sample size** | | **Standardized effect size (95% CI)** |
| --- | --- | --- | --- | --- | --- | --- | --- |
|  |  |  |  |  | **Smallest** | **Largest** |  |
| 1. TEA, thoracotomy | Joshi GP, Bonnet F, Shah R, Wilkinson RC, Camu F, Fischer B, Neugebauer EA, Rawal N, Schug SA, Simanski C, Kehlet H. A systematic review of randomized trials evaluating regional techniques for postthoracotomy analgesia. Anesth Analg. 2008 Sep;107(3):1026-40. | 17 | TEA with LA and opioids vs. systemic opioids | Postoperative 24 hr pain score at rest | 19 | 50 | -1.08^a^  (-1.61, -0.54) |
| 1. TEA, abdominal surgery | Perivoliotis K, Sarakatsianou C, Georgopoulou S, Tzovaras G, Baloyiannis I. Thoracic epidural analgesia (TEA) versus patient-controlled analgesia (PCA) in laparoscopic colectomy: a systematic review and meta-analysis. Int J Colorectal Dis. 2019 Jan;34(1):27-38. | 8 | TEA vs. PCA | Hospital LOS | 20 | 122 | 0.11^a^  (0.04, 0.19) |
| 1. ISB, shoulder surgery | Xiao M, Cohen SA, Cheung EV, Freehill MT, Abrams GD. Pain management in shoulder arthroplasty: a systematic review and network meta-analysis of randomized controlled trials. J Shoulder Elbow Surg. 2021;30(11):2638-2647. | 6 | Single-shot ISB vs. local liposomal bupivacaine injection | 4hr postoperative pain | 57 | 156 | -1.04  (-1.59, -0.48) |
| 1. Supraclavicular block, hand surgery | No reviews found |  |  |  |  |  |  |
| 1. Infraclavicular block, hand surgery | No reviews found |  |  |  |  |  |  |
| 1. Axillary block, hand surgery | No reviews found |  |  |  |  |  |  |
| 1. Pecs block, breast surgery | Elshanbary AA, Zaazouee MS, Darwish YB, et al. Efficacy and Safety of Pectoral Nerve Block (Pecs) Compared With Control, Paravertebral Block, Erector Spinae Plane Block, and Local Anesthesia in Patients Undergoing Breast Cancer Surgeries: A Systematic Review and Meta-analysis. Clin J Pain. 2021;37(12):925-939. | 25 | Pecs II vs. no block or sham block | Intraoperative opioid consumption | 39 | 255 | -1.75  (-2.66, -0.85) |
| 1. SAPB, breast surgery | Singh NP, Makkar JK, Kuberan A, Guffey R, Uppal V. Efficacy of regional anesthesia techniques for postoperative analgesia in patients undergoing major oncologic breast surgeries: a systematic review and network meta-analysis of randomized controlled trials. Can J Anaesth. 2022 Apr;69(4):527-549. | 4 | SAPB vs. no intervention | 24hr postoperative pain score at rest | 58 | 60 | N/A^b^ |
| 1. SAPB, VATS | De Cassai A, Boscolo A, Zarantonello F, et al. Serratus anterior plane block for video-assisted thoracoscopic surgery: A meta-analysis of randomised controlled trials. Eur J Anaesthesiol. 2021;38(2):106-114. | 7 | SAPB vs. general anesthesia with or without wound infiltration | Intraoperative opioid consumption | 40 | 85 | -0.62^a^  (-1.60, 0.36) |
| 1. PVB, breast surgery | Singh NP, Makkar JK, Kuberan A, Guffey R, Uppal V. Efficacy of regional anesthesia techniques for postoperative analgesia in patients undergoing major oncologic breast surgeries: a systematic review and network meta-analysis of randomized controlled trials. Can J Anaesth. 2022 Apr;69(4):527-549. | 16 | Single injection PVB vs. no intervention | 24hr postoperative pain score at rest | 40 | 378 | N/A^b^ |
| 1. PVB, thoracotomy | Balzani E, Rosboch GL, Ceraolo E, Lyberis P, Filippini C, Piccioni F, Guerrera F, Ruffini E, Pedoto A, Brazzi L. The effect of peripheral regional analgesia in thoracic surgery: a systematic review and a meta-analysis of randomized-controlled trials. Tumori. 2022 Epub ahead of print. | 2 | PVB vs. no intervention | Intraoperative opioid consumption | 61 | 63 | -0.77  (-1.38, -0.16) |
| 1. PVB, VATS | Jo Y, Park S, Oh C, Pak Y, Jeong K, Yun S, Noh C, Chung W, Kim YH, Ko YK, Hong B. Regional analgesia techniques for video-assisted thoracic surgery: a frequentist network meta-analysis. Korean J Anesthesiol. 2022 Jun;75(3):231-244. | 4 | PVB vs. control | 24hr postoperative opioid consumption | 47 | 60 | N/A^b^ |
| 1. PVB, abdominal surgery | Xuan C, Yan W, Wang D, Li C, Ma H, Mueller A, Deng H, Houle T, Wang J. Efficacy of different analgesia treatments for abdominal surgery: A network meta-analysis. Eur J Pain. 2022 Mar;26(3):567-577. | 5 | Preoperative single-shot PVB vs. placebo | 24hr postoperative pain score at rest | 34 | 58 | N/A^b^ |
| 1. ESPB, thoracotomy | Koo CH, Lee HT, Na HS, Ryu JH, Shin HJ. Efficacy of Erector Spinae Plane Block for Analgesia in Thoracic Surgery: A Systematic Review and Meta-Analysis. J Cardiothorac Vasc Anesth. 2022;36(5):1387-1395. | 6 | ESPB vs. no block | 24hr postoperative pain score at rest | 56 | 77 | -0.45^a^  (-0.70, -0.21) |
| 1. ESPB, VATS | Jo Y, Park S, Oh C, Pak Y, Jeong K, Yun S, Noh C, Chung W, Kim YH, Ko YK, Hong B. Regional analgesia techniques for video-assisted thoracic surgery: a frequentist network meta-analysis. Korean J Anesthesiol. 2022 Jun;75(3):231-244. | 8 | ESPB vs. control | 6hr postoperative pain score at rest | 40 | 90 | N/A^b^ |
| 1. ESPB, abdominal surgery | Viderman D, Aubakirova M, Abdildin YG. Erector Spinae Plane Block in Abdominal Surgery: A Meta-Analysis. Front Med (Lausanne). 2022;9:812531. | 6 | ESPB vs. no block | Opioid consumption within 24hr after surgery | 30 | 60 | -1.33  (-2.01, -0.65) |
| 1. ESPB, cardiac surgery^c^ | Hu M, Wang Y, Hao B, Gong C, Li Z. Evaluation of Different Pain-Control Procedures for Post-cardiac Surgery: A Systematic Review and Network Meta-Analysis. Surg Innov. 2022;29(2):269-277. | 1 | ESPB vs. target-controlled infusion | Postoperative pain score at rest | 106 | 106 | N/A^b^ |
| 1. TAP block, cesarean section | Ryu C, Choi GJ, Jung YH, Baek CW, Cho CK, Kang H. Postoperative Analgesic Effectiveness of Peripheral Nerve Blocks in Cesarean Delivery: A Systematic Review and Network Meta-Analysis. J Pers Med. 2022;12(4):634. | 11 | Lateral TAP block vs. control | 6hr postoperative pain score at rest | 40 | 134 | N/A^b^ |
| 1. TAP block, abdominal surgery | Viderman D, Aubakirova M, Abdildin YG. Transversus Abdominis Plane Block in Colorectal Surgery: A Meta-Analysis. Front Med (Lausanne). 2022;8:802039. | 5 | TAP block vs. no block | Opioid consumption within 24hr after surgery | 51 | 72 | -0.48  (-0.82, -0.14) |
| 1. TAP block, inguinal hernia repair | Grape S, Kirkham KR, Albrecht E. The analgesic efficacy of transversus abdominis plane block vs. wound infiltration after inguinal and infra-umbilical hernia repairs: A systematic review and meta-analysis with trial sequential analysis. Eur J Anaesthesiol. 2022 Epub ahead of print | 7 | TAP block vs. wound infiltration | 2hr postoperative pain score at rest | 40 | 71 | -0.65^a^  (-1.15, -0.15) |
| 1. QL (lateral/1) block, abdominal surgery | Uppal V, Retter S, Kehoe E, McKeen DM. Quadratus lumborum block for postoperative analgesia: a systematic review and meta-analysis. Can J Anaesth. 2020 Nov;67(11):1557-1575. | 5 | QL block vs. no block or placebo | 24hr postoperative pain score at rest^d^ | 48 | 74 | -0.08^a^  (-0.45, 0.28) |
| 1. RSB, abdominal surgery | Hamid HKS, Ahmed AY, Alhamo MA, Davis GN. Efficacy and Safety Profile of Rectus Sheath Block in Adult Laparoscopic Surgery: A Meta-analysis. J Surg Res. 2021 May;261:10-17. | 11 | RSB vs. control | 0-2hr postoperative pain score at rest | 38 | 61 | -1.83  (-2.70, -0.96) |
| 1. LPB, hip surgery | Højer Karlsen AP, Geisler A, Petersen PL, Mathiesen O, Dahl JB. Postoperative pain treatment after total hip arthroplasty: a systematic review. Pain. 2015 Jan;156(1):8-30. | 4 | LPB vs. control | Opioid consumption within 24hr after surgery | 30 | 150 | -0.39^a^  (-0.58, -0.21) |
| 1. Femoral or fascia iliaca block, hip surgery | Skjold C, Møller AM, Wildgaard K. Pre-operative femoral nerve block for hip fracture-A systematic review with meta-analysis. Acta Anaesthesiol Scand. 2020;64(1):23-33. | 5 | FNB vs. systemic analgesia | 0-2hr postoperative pain score at rest | 45 | 266 | -1.06^a^  (-1.76, -0.36) |
| 1. Femoral block, total knee arthroplasty | Liu Q, Wang A, Zhang J. The effects of local infiltration anesthesia and femoral nerve block analgesia after total knee arthroplasty: a systematic review and meta-analysis. Ann Transl Med. 2022;10(4):178. | 9 | FNB vs. local infiltration | Opioid consumption within 24hr after surgery | 36 | 120 | N/A^b^ |
| 1. ACB or saphenous block, total knee arthroplasty | Qin L, You D, Zhao G, Li L, Zhao S. A comparison of analgesic techniques for total knee arthroplasty: A network meta-analysis. J Clin Anesth. 2021;71:110257. | 16 | ACB vs. placebo | Postoperative pain score at rest | 40 | 155 | N/A^b^ |
| 1. IPACK block, total knee arthroplasty | Hussain N, Brull R, Sheehy B, Dasu M, Weaver T, Abdallah FW. Does the addition of iPACK to adductor canal block in the presence or absence of periarticular local anesthetic infiltration improve analgesic and functional outcomes following total knee arthroplasty? A systematic review and meta-analysis. Reg Anesth Pain Med. 2021 Aug;46(8):713-721. | 14 | iPACK with ACB vs. ACB alone | 6hr postoperative pain score at rest | 18 | 119 | -0.93^a^  (-1.10, -0.76) |
| 1. Popliteal sciatic block, foot/ankle surgery | No reviews found |  |  |  |  |  |  |
| 1. Ankle block, forefoot surgery/bunion | No reviews found |  |  |  |  |  |  |

ACB, adductor canal block; CI, confidence interval; ESPB, erector spinae plane block; FNB, femoral nerve block; ICNB, intercostal nerve block; IPACK, interspace between the popliteal artery and capsule of the knee; ISB, interscalene block; IV, intravenous; LA, local anesthesia; LPB, lumbar plexus block; LOS, length of stay; N/A, not available; PCA, patient-controlled analgesia; PVB, paravertebral block; QL, quadratus lumborum; RSB, rectus sheath block; SAPB, serratus anterior plane block; TEA, thoracic epidural analgesia; VATS, video-assisted thoracoscopic surgery

^a^ Weighted mean difference from original publication divided by pooled standard deviation to compute standardized effect size

^b^ Insufficient data presented in the original publication to convert weighted mean difference into a standardized effect size

^c^ Data not included in calculation of simulation inputs, due to inclusion of only 1 eligible trial in the meta-analysis.

^d^ Meta-analysis included 2 trials with lateral QL block, 2 trials with anterior QL block, and 1 trial with posterior QL block.
