# Supplementary material for: Simulation-Based Analysis of Trial Design in Regional Anesthesia
Source: Anesthesiol Res Pract. 2024 Mar 15;2024:6651894. doi: 10.1155/2024/6651894 (PMC10959581; doi:10.1155/2024/6651894)
Supplement: Supplementary Materials — Appendix Table 1: combinations of block type and indication included in search of regional anesthesia meta-analyses, and characteristics coded from each article. Appendix 2: data elements coded from each meta-analysis article. Appendix 3: Stata program to simulate sequence of randomized controlled trials. [file 6651894.f1.zip › Supplemental Appendix Table 2.docx]

**Appendix 2.** Data elements coded from each meta-analysis article.

| **Data element** | **Definition** | **Comments** |
| --- | --- | --- |
| Patient population | Patients included in the meta-analysis of the focal outcome. |  |
| Control | Placebo, no block, or sham block used as the comparison to the intervention | In studies with multiple control conditions, this was determined based on the first comparison described for the focal outcome. |
| Focal outcome | Continuous, quantitative outcome compared between an intervention and a control group in the meta-analysis. | Determined based on the eligible comparison mentioned in the results section of each article that was based on the largest number of trials. If the results section referred to a table summarizing multiple comparisons, the eligible comparison based on the largest number of trials was used. |
| Number of trials |  | Only trials contributing to the first comparison of the focal outcome were included in this count. |
| Sample size of smallest trial |  | Determined from trials contributing to the comparison of the focal outcome. |
| Sample size of largest trial |  | Determined from trials contributing to the comparison of the focal outcome. |
| Effect size (95% CI) | Standardized effect size for the focal outcome, based on the earliest comparison reported in the text. | If effect sizes were reported in unstandardized units (e.g. mg morphine equivalents), a pooled SD was calculated based on included trials, and used to convert to standardized effect sizes. |

CI, confidence interval; SD, standard deviation
